# Effect of Content of Sulfate Groups in Seaweed Polysaccharides on Antioxidant Activity and Repair Effect of Subcellular Organelles in Injured HK-2 Cells

**DOI:** 10.1155/2017/2542950

**Published:** 2017-07-12

**Authors:** Xiao-Tao Ma, Xin-Yuan Sun, Kai Yu, Bao-Song Gui, Qin Gui, Jian-Ming Ouyang

**Affiliations:** ^1^Department of Nephrology, The Second Hospital of Xi'an Jiaotong University, Xi'an 710004, China; ^2^Institute of Biomineralization and Lithiasis Research, Jinan University, Guangzhou 510632, China

## Abstract

This study aims to investigate the repair effect of subcellular structure injuries of the HK-2 cells of four degraded seaweed polysaccharides (DSPs), namely, the degraded *Porphyra yezoensis*, *Gracilaria lemaneiformis*, *Sargassum fusiform*, and *Undaria pinnatifida* polysaccharides. The four DSPs have similar molecular weight, but with different content of sulfate groups (i.e., 17.9%, 13.3%, 8.2%, and 5.5%, resp.). The damaged model was established using 2.8 mmol/L oxalate to injure HK-2 cells, and 60 *μ*g/mL of various DSPs was used to repair the damaged cells. With the increase of sulfate group content in DSPs, the scavenging activity of radicals and their reducing power were all improved. Four kinds of DSPs have repair effect on the subcellular organelles of damaged HK-2 cells. After being repaired by DSPs, the release amount of lactate dehydrogenase was decreased, the integrity of cell membrane and lysosome increased, the Δ*ψ*m increased, the cell of G1 phase arrest was inhibited, the proportion of S phase increased, and cell apoptotic and necrosis rates were significantly reduced. The greater the content of sulfate group is, the stronger is the repair ability of the polysaccharide. These DSPs, particularly the polysaccharide with higher sulfate group content, may be a potential drug for the prevention and cure of kidney stones.

## 1. Introduction

Oxidative stress is one of the main factors that cause diseases [1]. High concentration of oxalate and calcium oxalate crystal can cause the oxidative damage or dysfunction of renal epithelial cells and promote crystal retention [2], resulting in an excessive production of reactive oxygen species (ROS). The ROS induces the lipid peroxidation of biological membranes by reacting with unsaturated fatty acids on the cell membrane [3], disrupts structural integrity and energy production, and even causes cell necrosis, which will accelerate the formation of kidney stone [4]. Thus, the reduction of oxidative damage of cells and the repair of the damaged cells can reduce the incidence rate of kidney stones.

Sulfated seaweed polysaccharides exhibit antioxidant activities, which are closely related to their physicochemical properties, such as molecular weight, −OSO_3_H content, and polyphenol content [5–8]. For example, Wang et al. [7] extracted three components from *Laminaria japonica* (F1, F2, and F3) with sulfate contents of 23.30%, 36.41%, and 36.67%, respectively, and their ability of scavenging superoxide anion, hydroxyl radical, chelating ability, and reducing power all decreased in the order of F3 > F2 > F1, which suggested that sulfate content was positively related with the bioactivities of a polysaccharide. Imbs et al. [8] studied the antioxidant activities of fucose-containing sulfated polysaccharides (FCSPs) obtained from *Fucus evanescens*; the results showed that the antioxidant activity of FCSPs was positively correlated with polyphenol content in FCSPs.

Moreover, sulfated seaweed polysaccharides have protective and repair effects on damaged cells caused by oxidative stress [9–13]. For instance, sulfated polysaccharides extracted from *Sargassum horneri* can protect RAW264.7 cells against H_2_O_2_-induced oxidative injury [9]. *Stichopus japonicus* polysaccharide (SJP) can protect PC12 from Na_2_S_2_O_4_-induced oxidative damage [10]. The cell viability of the SJP protective group (64.7 ± 1.64%) was higher than the unprotected group (46.8 ± 1.4%); SJP can significantly decrease MDA level and increase SOD activity and mitochondrial membrane potential. Thevanayagam et al. [12] found that the repair effect of seaweed polysaccharide on the UV-induced normal human keratinocyte (HaCaT) cell damage gradually increased with the increasing sulfation degree of polysaccharide. Wang et al. [13] reported two sulfated *Cyclocarya paliurus* polysaccharides (S-CP_1–4_ and S-CP_1–8_) with a substitution degree of 0.42 ± 0.04 and 0.12 ± 0.02, respectively, and found that they had better protective effect on RAW264.7 cells against H_2_O_2_-induced oxidative stress as compared with the native polysaccharide. In addition, their protective effect was correlated with their sulfation degree.

In the previous report [14], four seaweed polysaccharides, namely, *Porphyra yezoensis* polysaccharide (DPY-1), *Gracilaria lemaneiformis* polysaccharide (DGL-2), *Sargassum fusiform* polysaccharide (DSF-3), and *Undaria pinnatifida* polysaccharide (DUP-4), were degraded by controlling the concentration of hydrogen peroxide and using four kinds of sulfation degree of polysaccharide products with similar molecular weight of about 3700 Da, but with different content of sulfate group (−OSO_3_H) consisting 17.9%, 13.3%, 8.2%, and 5.5%, respectively. The structure of these polysaccharides was studied, showing that the four seaweed polysaccharides are mainly composed of galactose and/or fucose. DPY-1 and DGL-2 are primarily composed of galactose; the contents of which reach to 92.0 and 95.8, respectively. Meanwhile, DSF-3 and DUP-4 mostly contained galactose and fucose [15–18]. Furthermore, we found that these polysaccharides were not toxic to human renal proximal tubular epithelial cells (HK-2) in the concentration range of 0–100 *μ*g/mL. Additionally, the repair effect of polysaccharides was determined using cell viability test by CCK-8 assay and cell morphology observation by hematoxylin-eosin staining, respectively. Results showed that the degraded polysaccharide had better repair effect than the original polysaccharide.

The functions of subcellular organelles changed after the cells were damaged. To further study the effect of −OSO_3_H content on the repair effect of seaweed polysaccharide, the repair effect of the above four degraded seaweed polysaccharides (DSPs) with different −OSO_3_H content on subcellular organelles of damaged HK-2 cells was investigated. We expect to explore the repair mechanism of DSPs at cellular and molecular levels, provide scientific basis for the prevention of kidney stone, and develop new antistone drugs.

## 2. Experiments

### 2.1. Reagents and Apparatus

Human kidney proximal tubular epithelial (HK-2) cells were purchased from Shanghai Cell Bank, Chinese Academy of Sciences (Shanghai, China). Dulbecco's modified Eagle's medium (DMEM) and fetal bovine serum were purchased from HyClone Biochemical Products Co. Ltd. (UT, USA). Cell culture plates of 6, 12, and 96-well were purchased from Wuxi Nest Bio-Tech Co. Ltd. (Wuxi, China). Trypsin, penicillin and streptomycin, 5,5′,6,6′-tetrachloro-1,1′,3,3′-tetraethylbenzimi-dazolylcarbocyanine iodide (JC-1), propidium iodide (PI), annexin V-FITC/PI, acridine orange (AO), and lactate dehydrogenase (LDH) kit were all purchased from Shanghai Beyotime Bio-Tech Co. Ltd.(Shanghai, China). Ethyl alcohol, oxalate, and other chemical reagents were all analytically pure and purchased from Guangzhou Chemical Reagent Factory of China (Guangzhou, China).

*Porphyra yezoensis* polysaccharide, *Gracilaria lemaneiformis* polysaccharide, *Sargassum fusiform* polysaccharide, and *Undaria pinnatifida* polysaccharides were produced by Beijing Newprobe Instrument Co. Ltd. Their degraded products with similar molecular weight (about 3700 Da), named as DPY-1, DGL-2, DSF-3, and DUP-4, were obtained by controlling the degradation condition (such as H_2_O_2_ concentration, degraded temperature, and degraded time) according to previous paper [14]. The contents of sulfate group and carboxyl group were shown in Table 1. The possible content of polyphenols mixed in the four different polysaccharides was determined by Folin-Ciocalteu method using gallic acid as a standard [19]. The results showed that the four polysaccharides do not contain polyphenols. Thus, the effect of polyphenol on the antioxidant activity of polysaccharides can be excluded.

The apparatus included enzyme mark instrument (Safire, Tecan, Switzerland), flow cytometry (FACS Aria, American BD Company), fluorescence microscope (Leica DMIRE2, Germany), optical microscope (Olympus, CKX41, Japan), and UV-Vis spectrophotometer (Cary 500, Varian Company, USA).

### 2.2. Antioxidant Activity Assays of Polysaccharides

#### 2.2.1. Hydroxyl Radical (·OH) Scavenging Activity of Polysaccharides

The ·OH scavenging ability of polysaccharide in vitro was detected by H_2_O_2_/Fe system method [20]. The reaction mixture that contained different concentrations of polysaccharides (0.15–3.0 mg/mL, 1 mL) was incubated with phenanthroline (2.5 mmol/L, 1 mL), ferrous sulfate (2.5 mmol/L, 1 mL), and hydrogen peroxide (20 mmol/L, 1 mL) in phosphate buffer (20 mmol/L, 1 mL, pH 7.4) for 90 min at 37°C. The absorbance measured at 536 nm was designated *A*_1_. The absorbance when hydrogen peroxide (H_2_O_2_) was replaced with distilled water and polysaccharide solution was *A*_2_ and *A*_3_, respectively. The ascorbic acid (Vc) was used as positive control group. The ability to scavenge hydroxyl radicals was calculated using the following equation:

(1) $$\begin{array}{c} Scavenging\;effect\;\left(\%\right)=\frac{\left(A_{3}-A_{1}\right)}{\left(A_{2}-A_{1}\right)}\times 100\%. \end{array}$$

#### 2.2.2. DPPH Radical Scavenging Activity of Polysaccharides

The DPPH radical scavenging activity was carried out according to Wang et al. [21] with minor modification. DPPH solution was prepared to be 0.4 mmol/L using dehydrated alcohol, each polysaccharide solution (3 mL) was mixed with DPPH solution (0.4 mmol/L, 1 mL) in a test tube, and final concentration of polysaccharide and DPPH solution was 0.15, 0.5, 0.8, 1.0, 2.0, 3.0 mg/mL, and 0.1 mmol/L, respectively. After the mixture was left to stand for 30 min at 25°C in the dark, the absorbance was measured at 517 nm. The ascorbic acid (Vc) was used as positive control group. The ability to scavenge DPPH radicals was calculated using the following equation:

(2) $$\begin{array}{c} Scavenging\;effect\;\left(\%\right)=\left[1-\frac{\left(A_{2}-A_{1}\right)}{A_{0}}\right]\times 100\%, \end{array}$$

where *A*_2_ was the absorbance of 3 mL sample mixed with 1 mL DPPH solution; *A*_1_ was the absorbance of 3 mL sample mixed with 1 mL blank solvent (dehydrated alcohol); and *A*_0_ was 1 mL DPPH solution mixed with 3 mL blank solvent.

DPPH is stable in the form of nitrogen free radical, and the solution is purple. When antioxidant was added, the antioxidant donated an electron or hydrogen to DPPH and formed a stable DPPH-H molecule and purpleness of the solution faded. Therefore, the DPPH free radical scavenging rate can be calculated through the change of absorbance.

#### 2.2.3. ABTS Radical Scavenging Activity of Polysaccharides

The ABTS radical scavenging activity of polysaccharides was performed according to [22] with slight modification. 7 mmol/L ABTS solution was mixed with 2.45 mmol/L potassium persulfate aqueous solution, and then, the mixture was incubated in the dark at room temperature for 12–16 h. Then, 3.0 mL mixture solution was added to 1 mL of various polysaccharide solutions (0.15–3 mg/mL) in test tube. After reacting for 6 min at room temperature, the absorbance was measured at 734 nm.

(3) $$\begin{array}{c} Scavenging\;effect\;\left(\%\right)=\left[1-\frac{\left(A_{1}-A_{2}\right)}{A_{0}}\right]\times 100\%, \end{array}$$

where *A*_0_ is the control group without polysaccharide; *A*_1_ is the experiment group; and *A*_2_ is the blank group without reagents (the absorbance of polysaccharide solution (*A*_2_) was 0).

#### 2.2.4. Reducing Power of Polysaccharides

The reducing power of polysaccharides was determined by referring to [23] with some modifications. 2.5 milliliter of polysaccharide samples in different concentrations (0.15–3.0 mg/mL) was mixed with 2.5 mL phosphate buffer (PBS, pH = 6.6) and 2.5 mL potassium ferricyanide (1.0%, *w*/*v*). The mixture was incubated at 50°C for 30 min and cooled to room temperature. 2.5 milliliter of trichloroacetic acid (10%, *w*/*v*) was added to the mixture which was then centrifuged for 10 min at 3000 r/min. The supernatant (2.5 mL) was mixed with 0.5 mL FeCl_3_·6H_2_O (0.1%, *w*/*v*) solution and 5.0 mL distilled water. The mixture was fully mixed and stood for 10 min. The absorbance was measured at 700 nm. The ascorbic acid (V_C_) was used as a positive control group and for comparison.

Antioxidants can donate an electron which reduce ferric of potassium ferricyanide (K_4_[Fe(CN)_6_]) to ferrous iron, and then, ferrous iron reacted with FeCl_3_·6H_2_O to form Fe_4_[Fe_6_(CN)_3_]_3_ with maximum absorbance value at 700 nm. Therefore, the absorbance measured at 700 nm can indirectly reflect reducing power. A higher absorbance indicated that stronger reducing power.

### 2.3. Cell Repair Assays of Polysaccharides with Different Content of Sulfate Groups

#### 2.3.1. Cell Culture

HK-2 cells were cultured in a DMEM culture medium containing 10% fetal bovine serum and 100 U/mL penicillin-100 *μ*g/mL streptomycin antibiotics with pH 7.4 at 37°C in a 5% CO_2_ humidified environment. Upon reaching 80%–90% confluent monolayer, cells were blown gently after trypsin digestion to form cell suspension for the following cell experiment.

#### 2.3.2. Lactate Dehydrogenase (LDH) Release Assay

Cell suspension with a cell concentration of 1 × 10^5^ cells/mL was inoculated per well in 96-well plates and incubated in DMEM culture medium for 24 h. The cells were divided into five groups: (1) cell-free culture medium wells (control wells of background); (2) control wells without drug treatment (sample control wells); (3) cells without drug treatment for the subsequent cleavage of the wells (sample maximum enzyme activity control wells); (4) injury group, the serum-free medium containing 2.8 mmol/L oxalate was added and incubated for 3 h; (5) repair group, the serum-free medium containing 60 *μ*g/mL DPY-1, DGL-2, DSF-3, and DUP-4 polysaccharide was added to repair the damaged cells and incubated for 12 h. After the repair was completed, enzyme mark instrument was used to detect the OD value of each group according to LDH kit method. The results were calculated as LDH release amount (%) = (the absorbance of sample − treated group − the absorbance of sample control group)/(the absorbance of sample maximum enzyme activity − the absorbance of sample control group) × 100.

#### 2.3.3. Measurement of Mitochondria Membrane Potential (Δ*Ψ*m)

Cell suspension with a cell concentration of 1 × 10^5^ cells/mL was inoculated per well in 6-well plates and incubated as mentioned above. The cells were divided into three groups: (1) control group, (2) injury group, and (3) repair group; the repaired time was 12 h. After the repair was completed, the cells were collected and centrifuged at 1000 rpm/min for 5 min. After that, the supernatant was removed by suction and cells were rinsed twice with PBS. The Δ*Ψ*m was detected according to JC-1 kit. Then, the cells were stained with 200 *μ*L JC-1 dye, thoroughly mixed, and incubated in darkness at 37°C for 15 min. After treatment, the cells were detected by flow cytometry.

#### 2.3.4. Cell Cycle Assay [24]

Cell suspension with a cell concentration of 1 × 10^5^ cells/mL was inoculated per well in 6-well plates and incubated as mentioned above. After being repaired for 12 h, the cells were collected with trypsin digestion. The collected cells were washed twice with PBS and centrifugation (1000 rpm, 5 min), then fixed using 70% ethanol for 12 h at 4°C. Ethanol was removed by centrifugation (2000 rpm, 5 min), and the cells were washed twice with PBS. Cells were then resuspended in 200 *μ*L propidium iodide and kept at 37°C for 15 min. The cell cycle was analyzed by measuring the amount of PI–labeled DNA in fixed cells by the flow cytometer.

#### 2.3.5. Lysosomal Integrity Assay [25]

For fluorescence qualitative observation by fluorescence microscope, the cell suspension with a cell concentration of 1 × 10^5^ cells/mL was inoculated to subconfluence in 12-well plates with coverslips for 24 h. The cells were washed twice with PBS and then loaded with 5 *μ*g/mL AO in DMEM for 15 min. The cells were divided into three groups as mentioned above. After being repaired for 12 h, the cells were rinsed three times with PBS and the distribution of AO in cells was observed under fluorescence microscope.

For fluorescence quantitative detection by microplate reader, cells (1.0 × 10^5^ cells/mL) were cultured in a 96-well plate (100 *μ*L/well) and were stained with AO; the cells were washed with PBS before fluorescence measurements with excitation at 485 nm and emission at 530 (green cytoplasmic AO) and 620 nm (red lysosomal AO). Normal lysosomal integrity = (total red fluorescence intensity of normal lysosome)/(total green fluorescence intensity of normal lysosome). Lysosomal integrity = (total red fluorescence intensity)/[(total green fluorescence intensity) × (normal lysosomal integrity)].

#### 2.3.6. Cell Apoptosis Assay [26]

Cell suspension with a cell concentration of 1 × 10^5^ cells/mL was inoculated per well in 6-well plates and incubated for 24 h. As above, the cells were divided into three groups. After reaching the repair time of 12 h and doing corresponding treatment, the cells were collected and centrifuged at 1000 rpm/min for 5 min. The cells were resuspended in 200 *μ*L binding buffer. Afterward, 5 *μ*L annexin V-FITC was added and then incubated in darkness at room temperature for 10 min. The cells were again resuspended in 200 *μ*L binding buffer and stained with 5 *μ*L PI. The prepared cells were then analyzed using a flow cytometer.

### 2.4. Statistical Analysis

Experimental data were expressed by mean ± standard deviation ($\overset{¯}{x}$±SD). The experimental results were analyzed statistically using SPSS 13.0 software. The differences of means between the experimental groups and the control group were analyzed by Tukey. If *p* < 0.05, there was significant difference; if *p* < 0.01, the difference was extremely significant; if *p* > 0.05, there was no significant difference.

## 3. Results and Discussion

### 3.1. Antioxidant Activity of Polysaccharides with Different Sulfate Group Content

#### 3.1.1. Hydroxyl Radical (·OH) Scavenging Capacity

In biological ROS, ·OH is the most active radical, which can easily cross cell membranes, readily react with most biomolecules (including carbohydrates, proteins, lipids, and DNA in cells), and cause tissue damage or cell death, eventually leading to many diseases [27, 28].

**Figure 1:**
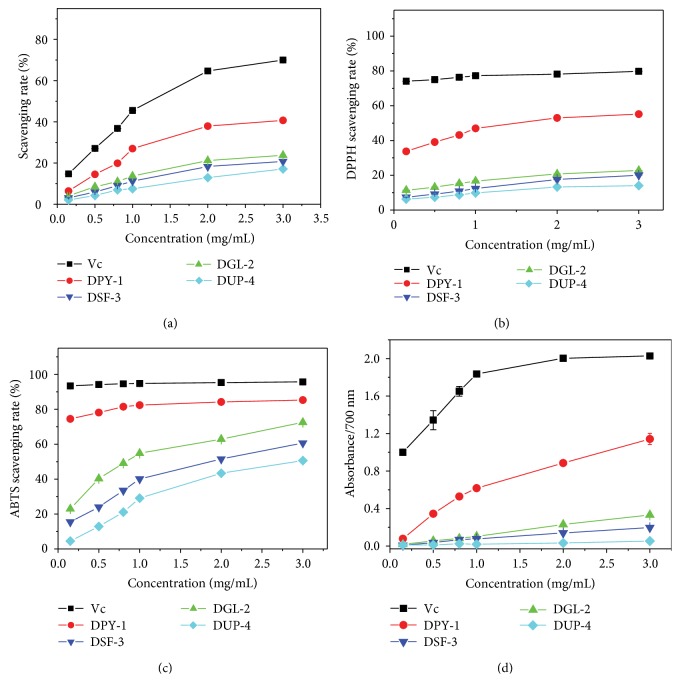
Comparison of antioxidant activity of four DSPs with different −OSO_3_H content: (a) hydroxyl radical scavenging rate; (b) DPPH radical scavenging rate; (c) ABTS radical scavenging rate; and (d) reducing power.

As shown in Figure 1(a), four DSPs showed the scavenging capacity of ·OH in a concentration-dependent manner. Results revealed that polysaccharides with higher concentration exerted stronger scavenging capacity. Also, the polysaccharide with higher sulfate group content has stronger ·OH scavenging capacity at the same concentration. For example, at the concentration of 3.0 mg/mL of a polysaccharide, the ·OH scavenging rate was DPY-1 (40.7%) > DGL-2 (23.8%) > DSF-3 (20.8%) > DUP-4 (17.1%), which was in accordance with the sequence of sulfate group content of four DSPs (17.9%, 13.3%, 8.2%, and 5.5%). The IC_50_ values of the four DSPs and Vc were 2.75, 7.21, 8.33, 9.21, and 1.57 mg/mL, respectively (Table 1).

#### 3.1.2. DPPH Radical Scavenging Capacity

The result of DPPH radical scavenging of four DSPs is shown in Figure 1(b). All the polysaccharides exhibited the DPPH radical scavenging capacity in a concentration-dependent manner, and a higher concentration of polysaccharide indicated a higher radical scavenging rate. For the different DSPs, the higher sulfate group content of polysaccharide indicated a stronger DPPH free radical scavenging capacity at the same concentration. For all the polysaccharides with similar molecular weight, the active −OSO_3_H group is the main factor affecting the free radical scavenging capacity of a polysaccharide. The IC_50_ values of scavenging DPPH free radical of the four DSPs and Vc were 14.6, 9.68, 8.24, 1.82, and 0.91 mg/mL, respectively (Table 1).

#### 3.1.3. ABTS Radical Scavenging Capacity

ABTS assay is an important method used to measure the antioxidant capacity of antioxidants. The result of ABTS radical scavenging of the four DSPs is shown in Figure 1(c). All the polysaccharides exhibited the ABTS radical scavenging capacity in concentration-dependent manner, and the higher concentration of polysaccharide indicated a higher radical scavenging rate. For the different DSPs, the higher sulfate group content of polysaccharide indicated a stronger ABTS free radical scavenging capacity at the same concentration. For all the polysaccharides with similar molecular weight, the active −OSO_3_H group is the main factor affecting the ABTS radical scavenging capacity of a polysaccharide.

#### 3.1.4. Reducing Power of Polysaccharide

The reducing power of a polysaccharide has a direct, positive correlation with antioxidant capacity [29]. Natural antioxidants can terminate free radical chain reaction by their capacity to donate an electron or hydrogen atom to free radicals. Thus, the reducing capacity of antioxidant may serve as a significant indicator of its potential antioxidant activity [30].

As shown in Figure 1(d), the absorbance measured at 700 nm increased with the increasing concentration of a polysaccharide, suggesting that the reducing power was strengthened in a concentration-dependent manner. For the different polysaccharides, the absorbance, that is, the reducing power of polysaccharide, increased with the increasing content of sulfate groups.

Antioxidant activity of polysaccharides is related to several structural characteristics, such as branch chain, glycosidic linkage, steric conformation, and monosaccharide composition; of which, the influences of molecular weight [31, 32] and acid group content [33, 34] are the most noticeable. For polysaccharides with similar molecular weight (approximately 3700 Da) in our study, the acid group content is the most critical factor that influences the antioxidant activity of polysaccharides. Comprehensively concluding the four antioxidant results in Figure 1, the antioxidant activity of DSPs has the following change rules:

1. Acidic groups in polysaccharide (such as −OSO_3_H) can be complex with transition metal ions (such as Fe^3+^) that are necessary in catalyzing the free radical chain reaction to stop the initiation of the free radical chain reaction and avoid the generation of radical ions. In addition, the sulfate group content is positively related to the antioxidant activity of DSPs. The electron-withdrawing sulfate groups of a polysaccharide can activate the hydrogen atom of sugar residue through field and inductive effects [13, 20] and donate hydrogen atom or an electron to a radical, which result in the termination of the radical chain reaction.
2. All the polysaccharides exhibited antioxidant activities in a concentration-dependent manner.

### 3.2. Repair Effect of Polysaccharides on Cell Membrane

LDH is a stable enzyme of the cytoplasm that is released extracellularly once the cell membrane ruptures. Thus, LDH is considered a marker of cell membrane integrity [35].

**Figure 2:**
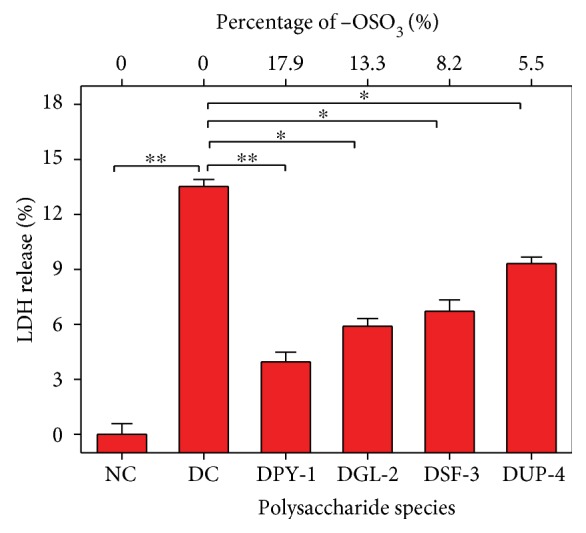
LDH release amount of the damaged HK-2 cells after exposure to four DSPs with different −OSO_3_H content. The concentration of oxalate to injury HK-2 cells: 2.8 mmol/L; injury time: 3 h; DSP concentration: 60 *μ*g/mL; repaired time: 12 h. NC: normal control; DC: damaged control. ∗∗ indicates *p* < 0.01; ∗ indicates *p* < 0.05.

The change of LDH release amount of the damaged HK-2 cells after being repaired by the four DSPs is shown in Figure 2. Compared with the damaged cells, LDH release amount of all the repair groups reduced in different degrees, and the reduced amount was positively correlated with the content of −OSO_3_H groups in polysaccharides. For example, after being repaired by DPY-1 with the highest content of −OSO_3_H (17.9%), the LDH release amount of the damaged HK-2 cells was the smallest (3.96%) as compared with 13.3% from the damaged group. The LDH release amount of the other three repaired groups was 5.90% to 9.31%. This finding suggested that DSPs with higher content of −OSO_3_H groups had better repair effect on the cell membrane of damaged HK-2 cells.

Numerous studies have reported the repair effect of polysaccharides on injured cell membranes. Hu et al. [36] studied the therapeutic effect of *Inonotus obliquus* polysaccharide (IOP) on the diethyldithiocarbamate- (DDC-) induced pancreatic acinar atrophy and chronic pancreatitis (CP) in mice. They found that the LDH release level (580 U/L) after treatment with IOP was significantly lower than the DDC-damaged group (880 U/L). Wen et al. [9] studied the protective effect of SHS against the H_2_O_2_-induced oxidative damage of RAW264.7 cell; the LDH release amount (82, 84, 105 U/L) was significantly reduced compared with the damaged group (145 U/L) after pretreated by SHS*_c_*, SHS_1_, and SHS_0.5_ polysaccharides, which had 4.95%, 4.48%, and 2.95% of −OSO_3_H content, respectively. The LDH release amount was smallest after the damaged HK-2 cells were repaired by SHS*_c_*, the polysaccharide that had the highest −OSO_3_H content.

### 3.3. Repair Effect of Polysaccharides on Mitochondria

A symbolic event in early apoptosis is the decrease in mitochondrial membrane potential (Δ*Ψ*m). JC-1 differentially labels mitochondria with high and low Δ*Ψ*m by forming J-aggregates or monomers that emit orange-red or green light, respectively [37]. The Δ*Ψ*m and concentration of J-aggregates were high in the mitochondria of living cells. From the degree that JC-1 changes from red to green fluorescence, the reduced amount of Δ*Ψ*m can be detected.

**Figure 3:**
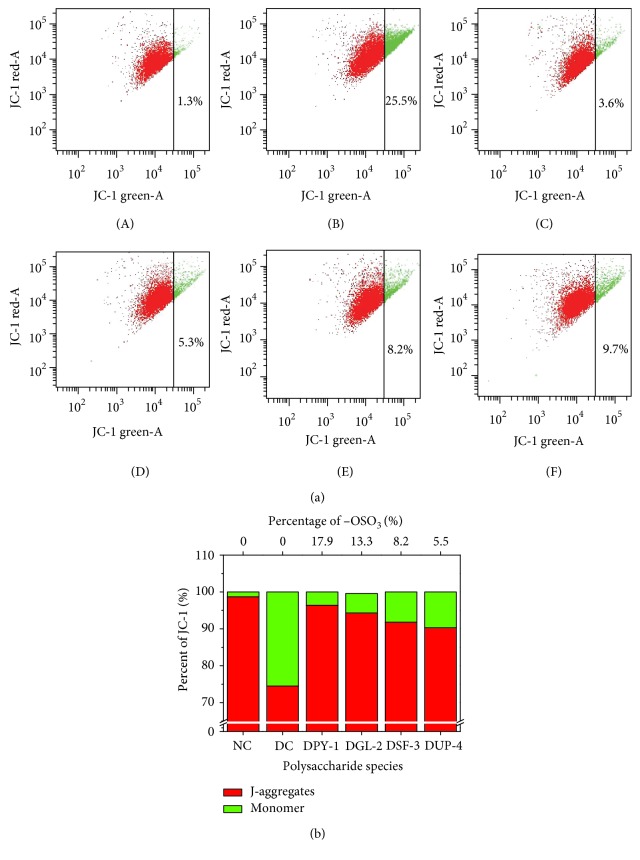
Mitochondrial membrane potential change after the damaged HK-2 cells were repaired by four DSPs with different −OSO_3_H content. (a) Dot plots of Δ*ψ*m change; (b) the aggregated degree of JC-1. JC-1 monomers represented the reduced Δ*ψ*m. (A) normal control; (B) damaged control; (C) DPY-1; (D) DGL-2; (E) DSF-3; (F) DUP-4. DSP concentration: 60 μg/mL; the damage concentration of oxalate: 2.8 mmol/L; injury time: 3 h; repaired time: 12 h.

The Δ*Ψ*m of HK-2 cell at different states is shown in Figure 3. The proportion of green fluorescence in the mitochondria of normal HK-2 cell was very low (1.3%) and obviously increased after the cell was damaged by oxalate (25.5%), which suggested that the Δ*Ψ*m decreased obviously. However, after the damaged cells were repaired by different polysaccharides, green fluorescence reduced with different degrees, and the reduced degree was positively correlated with the −OSO_3_H content of each polysaccharide. That is, the higher the −OSO_3_H content of a polysaccharide, the lesser green fluorescence and the higher repair degree of the Δ*Ψ*m become. In degraded DPY-1, which had the highest content of −OSO_3_H (17.9%) repaired group, the proportion of green fluorescence was lowest (3.6%).

Polysaccharides can protect the intracellular mitochondria from oxidative damage. Cui et al. [38] studied the protective effect of sulfated polysaccharide (such as SJP) against 6-OHDA-induced SH-SY5Y cell death and found that the Δ*Ψ*m of SJP pretreatment group (79%) was higher than 6-OHDA damaged group (56%), suggesting that SJP had certain protective effect on 6-OHDA-induced SH-SY5Y cell damage. Li et al. [39] examined the endothelial cell death mode caused by high glucose-induced ROS and suggested that *Ganoderma atrum* polysaccharide (PSG-1) had protective effect on the Δ*Ψ*m. The Δ*Ψ*m (36.26%) of high glucose-treated group was obviously lower than the cell of the control group (80.44%), but the Δ*Ψ*m increased (60.03%) after the cells were pretreated by PSG-1.

After the mitochondria were damaged, Δ*Ψ*m reduced and cytochrome c was released from the mitochondria to cytosol, which caused cell apoptosis [40]. Geng et al. [41] studied the effect of SMP1 on H_2_O_2_-induced cardiac muscle cell apoptosis after the cells were treated with H_2_O_2_ for 24 h. They found that the amount of cytochrome c in the mitochondria was significantly reduced, whereas the amount of cytochrome c released into the cytosol was significantly increased. They also found that the downregulation of Bcl-2 and upregulation of Bax expression was contrary to the results in SMP1 pretreatment group. Moreover, antiapoptotic Bcl-2 protein can inhibit cell apoptosis by preventing the release of cytochrome c from the mitochondria, whereas proapoptotic Bax protein can cause the release of cytochrome c into the cytosol by inducing the permeabilization of the mitochondrial membrane [42].

### 3.4. Repair Effect of Polysaccharides on Cell Cycle

**Figure 4:**
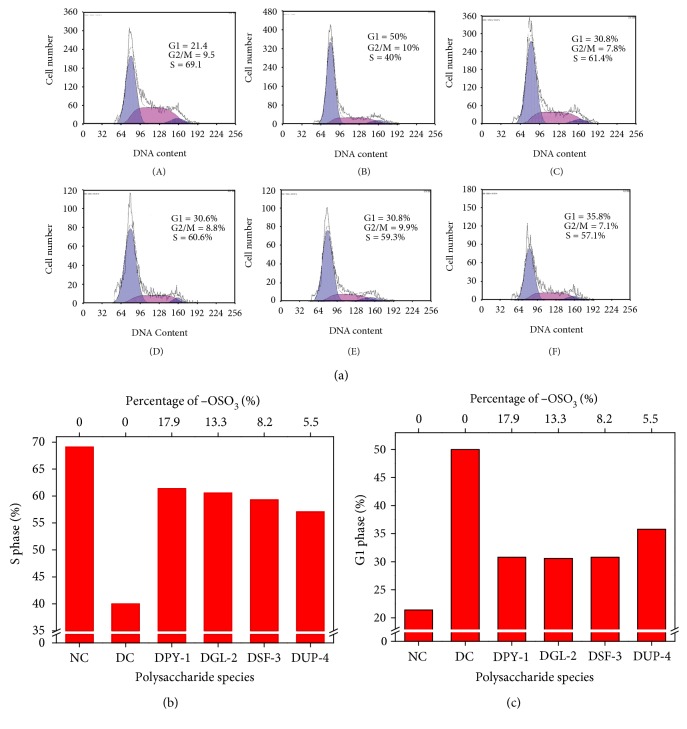
Cell cycle change of the damaged HK-2 cells after repair by four DSPs with different −OSO_3_H content. (a) Representative images of cell cycle; (b) S phase changes; (c) G1 phase changes. (A) normal control; (B) damaged control; (C) DPY-1; (D) DGL-2; (E) DSF-3; (F) DUP-4.

The combination of PI with double-stranded DNA can produce fluorescence, and the DNA content and distribution can be detected by flow cytometry; hence, the cell cycle can be analyzed. The cell cycle changes of each group are shown in Figure 4. The cell number of S phase (40%) was significantly reduced than that in the control group (69.1%) (Figure 4(b)); however, the cell number of G1 phase was markedly increased from 21.4% of the control group to 50.1% of the damaged group (Figure 4(c)). After the damaged HK-2 cells were repaired by DSPs, the cell proportion of S phase was significantly increased than the damaged group (Figure 4(b)), whereas the cell proportion of G1 phase was reduced. In detail, the cell proportion of S phase increased from 40% of the damaged group to 57.1%, 59.3%, 60.6%, and 61.4% of DPY-1, DGL-2, DSF-3, and DUP-4, respectively. That is, the higher the −OSO_3_H content of a polysaccharide, the larger the cell proportion of the S phase is; thus, the polysaccharide had greater repair effect on the damaged cell.

Pu et al. [43] showed that the cell proportion of the S phase (13.56%) was obviously reduced, whereas the cell proportion of the G1 phase (85.20%) was obviously greater compared with normal control cells with S phase fraction of 21.82% and G1 phase fraction of 61.94% after the WI-38 cells were treated with H_2_O_2_. This result suggested that H_2_O_2_ resulted in significant senescence symptom of WI-38 cell (G1 phase block). However, the cell proportion of G1 phase (36.36%) was significantly reduced, whereas the cell proportion of S phase (57.5%) increased in the Guiqi polysaccharide (GQP) pretreatment group. Ding et al. [44] showed that once the HELF cells were exposed to t-BHP (400 *μ*M), the G1 cell population (56.80%) was significantly higher, whereas the S cell population (39.29%) was lower compared with the normal control group with G1 and S phase fractions of 33.41% and 64.37%, correspondingly. Meanwhile, the G1 cell population (44.10%) was reduced and S cell population increased (53.45%) after the HELF cells were exposed to TLH-3.

After the normal cells were damaged, cells started self-detection and made the progression of cell cycle slow or temporarily blocked to prevent DNA from being replicated and to ensure that the cell genetic material was correct and reproduced accurately [45]. The self-repaired cell can go into the next stage, whereas the unrepaired cell arrested at G0/G1 phase and failed into S phase, which can make cells keep their normal biological function. Therefore, the cell proportion of G0/G1 phase increased, whereas the cell proportion of S phase reduced [44].

### 3.5. Repair Effect of Polysaccharides on Lysosome

Acridine orange (AO) is a weak base that can go into the lysosome through the cell membrane. AO differentially labels with high and low concentration by forming two polymers or monomers that emit red or green fluorescence. When the integrity of lysosome was destructed, the internal substance of lysosome leaked and orange-red fluorescence reduced [46]. Therefore, the repair degree of lysosome of cells can be determined by measuring the ratio of red and green fluorescence intensities.

**Figure 5:**
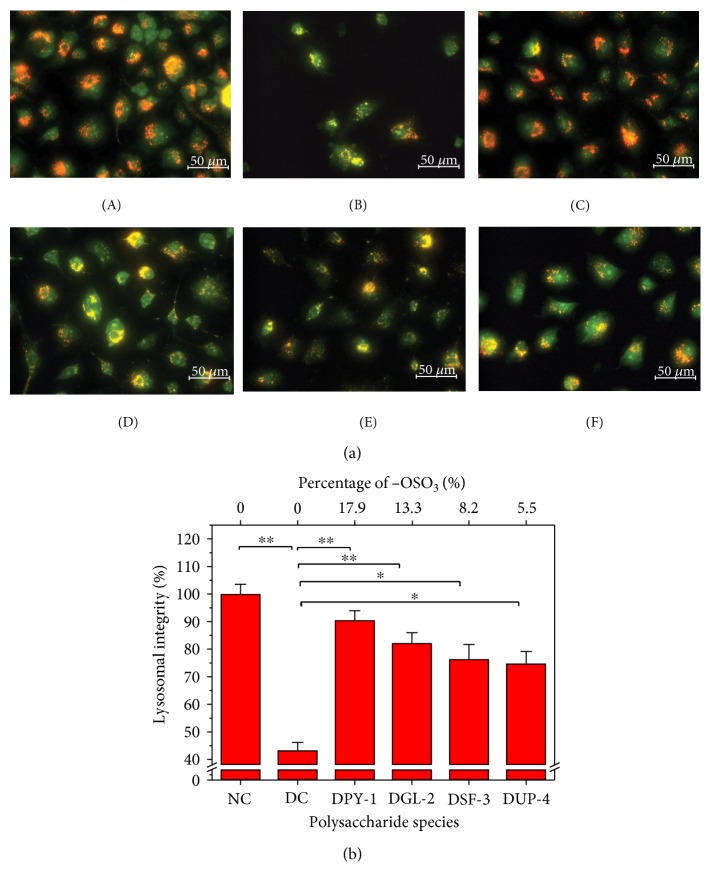
Change of lysosomal integrity after the damaged HK-2 cells were repaired by four DSPs with different −OSO_3_H content. (a) Fluorescence microscope observation; (b) quantitative analysis. Red fluorescence represented unbroken lysosomes; (A) normal control; (B) damaged control; (C) DPY-1; (D) DGL-2; (E) DSF-3; (F) DUP-4. DSP concentration: 60 μg/mL; the damage concentration of oxalate: 2.8 mmol/L injury time: 3 h; repaired time: 12 h. ∗∗ indicates *p* < 0.01; ∗ indicates *p* < 0.05.

The changes of lysosome integrity of HK-2 cell of each group are shown in Figure 5. The lysosome structure was complete, and the superposition of red and green fluorescence showed strong orange-red (100%) in normal control cells. When the normal cells were damaged by oxalate, red fluorescence was significantly reduced (43.29%). However, after the damaged cells were repaired by DSPs, red fluorescence intensity increased with different degrees (Figure 5(b)), and the degree of increase was positively correlated with the −OSO_3_H content of the polysaccharides. The red fluorescence intensity of DPY-1 that had the highest content of −OSO_3_H (17.9%) repair group was 90.50%. With the −OSO_3_H content of polysaccharides reduced to 13.3%, 8.2%, and 5.5%, the red fluorescence intensity of these repaired groups was 82.22%, 76.40%, and 74.75%, respectively. The above results suggested that DSPs had repair effect on lysosomes of cells.

Yue et al. [47] studied the protection effect of *Astragalus* polysaccharide (APS) on skin fibroblasts and found that the red fluorescence in cells was significantly reduced than in normal control cells after the skin fibroblasts were treated with 0.5 mmol/L H_2_O_2_ for 30 min. However, the red fluorescence in cells of APS repair group was increased. Rodrigues et al. [48] also studied the protective effect of *Aloe vera* (AV) extract on UVA-induced HaCaT lysosomal membrane and found that the lysosomal integrity of AV extract treatment group (82%) was obviously higher than that in the UVA-damaged group (45%), which suggested that lysosome can maintain its intrinsic ability of trapping proton after the AV was added.

### 3.6. Effect of Polysaccharide Preprotection on Cell Apoptosis

To assess the nature of crystal-induced cell death, we performed flow cytometric analysis to quantify the apoptotic and necrotic cells using annexin V/PI double staining. Annexin V staining was applied to reveal the surface exposure of phosphatidylserine (apoptosis), whereas PI was applied to reveal the loss of plasma membrane integrity (necrosis).

**Figure 6:**
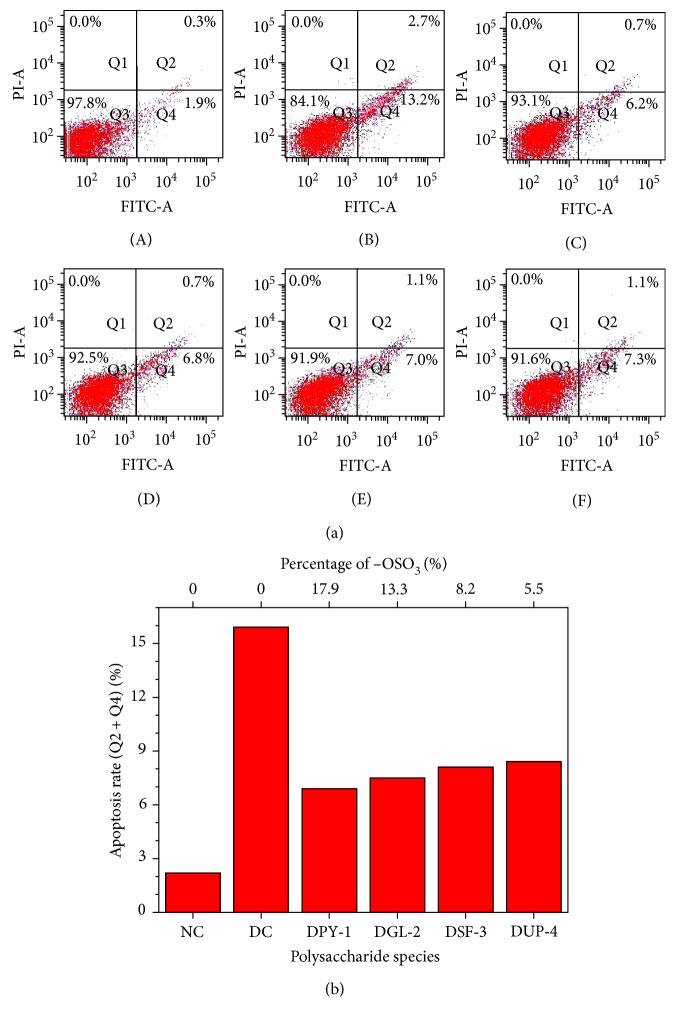
Cell apoptosis detected by flow cytometry after the damaged HK-2 cells were repaired by DSPs with different −OSO_3_H content. (a) Dot plots of cell apoptosis and necrosis; (b) the change of cell apoptosis rate (Q2 + Q4). (A) normal control; (B) damaged control; (C) DPY-1; (D) DGL-2; (E) DSF-3; (F) DUP-4.

The changes in the cell apoptotic rate of each group cell are shown in Figure 6. The total cell apoptotic rate (Q2 + Q4) in the normal control group was very low (2.2%), whereas the total cell apoptotic rate in the oxalate-damaged group was significantly increased (15.9%). After the damaged cells were repaired by different polysaccharides, the apoptotic rate of cells was decreased in different degrees (Figure 6(b)), and the degree of reduction was positively correlated with the content of −OSO_3_H of each polysaccharide. That is, the −OSO_3_H contents of DPY-1, DGL-2, DSF-3, and DUP-4 were 17.9%, 13.3%, 8.2%, and 5.5%, respectively, and the cell apoptotic rates of the repaired groups were 6.9%, 7.5%, 8.1%, and 8.4%, correspondingly.

Li et al. [39] examined the high glucose-induced ROS production and cell apoptosis of endothelial cells and suggested that the cell apoptotic rate of high glucose treatment group was 20.66%, whereas the cell apoptotic rate reduced to 12.68% after the cells were pretreated with PSG-1, which indicated that PSG-1 can alleviate cell apoptosis induced by oxidative stress. Similarly, Chowdhury et al. [49] revealed that the bacterial fucoidan can inhibit the H_2_O_2_-induced stress of human lung fibroblasts, and the total cell apoptotic rate of H_2_O_2_-damaged group and polysaccharide repair group was 42.6% and 10.0%, respectively.

## 4. Conclusions

Four types of DSPs (DPY-1, DGL-2, DSF-3, and DUP-4), with molecular weights of approximately 3700 Da and −OSO_3_H content of 17.9%, 13.3%, 8.2%, and 5.5%, respectively, were used to repair the HK-2 cells damaged by 2.8 mmol/L oxalate. With the increase of sulfate group content in DSPs, the scavenging activity of hydroxyl, DPPH, and ABTS radicals and their reducing power were all improved. The sulfate group content is positively related to the antioxidant activity of DSPs. After the damaged HK-2 cells were repaired by 60 *μ*g/mL polysaccharide, LDH release amount reduced, mitochondrial membrane potential improved, cell proportion of G1 phase reduced but cell proportion of S1 phase increased, lysosome integrity improved, and cell apoptotic rate reduced. Moreover, the higher the −OSO_3_H content of DSPs, the more obvious the repair effect of DSPs on various subcellular organelles of damaged HK-2 cells. These DSPs, especially for the polysaccharide with higher −OSO_3_H content, may become potential drugs for the prevention and cure of kidney stones.

## Figures and Tables

**Table 1:** Molecular weight and content of −OSO_3_H and −COOH groups of four DSPs as well as comparison of the scavenging capacity of ·OH and DPPH radicals.

Polysaccharide type	Mean molecular weight *M*/Da	−OSO_3_H content/%	−COOH content/%	·OH radical scavenging capacity IC_50_/mg/mL	DPPH radical scavenging capacity IC_50_/mg/mL
DPY-1	4020	17.9	1.7	2.75	1.82
DGL-2	3343	13.3	1.0	7.21	8.24
DSF-3	3828	8.2	1.3	8.33	9.68
DUP-4	3635	5.5	1.2	9.21	14.6
Vc control	—	—	—	1.57	0.91

DPY-1: degraded *Porphyra yezoensis* polysaccharide; DGL-2: degraded *Gracilaria lemaneiformis* polysaccharide; DSF-3: degraded *Sargassum fusiform* polysaccharide; DUP-4: degraded *Undaria pinnatifida* polysaccharide; Vc: ascorbic acid.
